# Effects of Continuous Chelation with Etidronate on Mechanical Properties of the Root Canal Dentin: A Systematic Review with Meta-Analysis

**DOI:** 10.3390/dj14070450

**Published:** 2026-07-18

**Authors:** Margarita Sachkova, Daria Savochkina, Nina Novozhilova, Ksenia Babina, Anna Mikheikina, Vladlena Doroshina, Alexandr Zaytsev, Maria Polyakova

**Affiliations:** 1 Department of Therapeutic Dentistry, Sechenov First Moscow State Medical University (Sechenov University), 119991 Moscow, Russiasavochkina_d_m@student.sechenov.ru (D.S.); novozhilova_n_e@staff.sechenov.ru (N.N.); mikheykina_a_m@staff.sechenov.ru (A.M.); doroshina_v_yu@staff.sechenov.ru (V.D.); polyakova_m_a_1@staff.sechenov.ru (M.P.); 2 Institute of Foreign Languages for Professional Purposes, Sechenov First Moscow State Medical University (Sechenov University), 119991 Moscow, Russia; zaytsev_a_b@staff.sechenov.ru

**Keywords:** dentin, etidronic acid, edetic acid, root canal irrigants, hardness, tensile strength, systematic review, meta-analysis

## Abstract

**Background**: Continuous chelation with etidronic acid (1-Hydroxyethylidene-1,1-diphosphonic acid, HEDP) has been proposed as a less aggressive alternative to sequential irrigation with ethylenediaminetetraacetic acid (EDTA) in endodontics. This systematic review and meta-analysis aimed to compare the effects of these two protocols on the mechanical properties and structure of root dentin. **Methods**: PubMed, Scopus, and Cochrane Central databases were searched up to June 2026 for in vitro studies comparing continuous chelation (NaOCl + HEDP mixture) with sequential irrigation (NaOCl and EDTA) on root dentin of permanent teeth. Studies on coronal dentin and those not using NaOCl + HEDP as a mixture were excluded. Risk of bias was assessed using the QUIN tool. Meta-analyses were performed for fracture resistance using a random-effects model. This review was registered in PROSPERO (CRD420250630663). **Results**: Twenty studies with a total of 572 specimens were included. Six studies had a low risk of bias and thirteen had a medium risk. The qualitative synthesis showed that HEDP generally resulted in similar or lower levels of erosion, microhardness reduction, demineralization, and collagen degradation compared to EDTA. The meta-analysis revealed no significant difference in fracture resistance (5 studies; SMD = 0.28; 95% CI: −0.37 to 0.92; *p* = 0.40); however, substantial heterogeneity was observed (I^2^ = 66.7%). **Conclusions**: The evidence was limited by substantial heterogeneity and mainly medium risk of bias. Within the limitations, in vitro evidence suggests that continuous chelation with HEDP is comparable to EDTA in terms of root dentin mechanical properties and composition, supporting its use as a chelating agent in endodontic practice. This research received no external funding.

## 1. Introduction

A vertical root fracture is defined by the American Association of Endodontics as “a fracture in the root whereby the fractured segments are incompletely separated” [1]. The prevalence of root fractures remains relatively high, ranging from 2% to 25% and, in some studies, reaching up to 32% [2,3,4]. This condition occurs predominantly in root-filled teeth, with mandibular molars and maxillary premolars being the most frequently affected due to the specific anatomic characteristics of their roots and the surrounding bone [2,4]. Risk factors related to previous endodontic treatment include dentin thinning during root canal preparation or post space preparation [5,6,7], the use of aggressive chemicals for root canal [8,9], and excessive forces applied during root canal obturation [10]. Age is considered a predisposing factor as well due to microstructural changes in hard tooth tissues [11,12].

The principal aim of root canal therapy is to eliminate microorganisms from the root canal and to prevent their invasion into periapical tissues through the thorough removal of pulp tissue remnants and antiseptic treatment [13,14]. Endodontic treatment implies access cavity creation, chemo-mechanical preparation of the root canal system, and three-dimensional obturation [14]. The quality of root canal instrumentation and irrigation is one of the key factors for endodontic treatment success [15].

Up-to-date irrigation protocols focus on the elimination of microorganisms and the removal of both organic and inorganic components of the smear layer from the root canal walls [16]. The most widely accepted endodontic irrigant is sodium hypochlorite (NaOCl), which is an effective antimicrobial and organic tissue-dissolving agent [17]. However, it should be combined with a chelating agent capable of dissolving the inorganic components of the smear layer [18,19,20], e.g., with ethylenediaminetetraacetic acid (EDTA). Although NaOCl and EDTA are the most common irrigants in endodontics [21], their use has notable drawbacks. Alternating irrigation with these solutions decreases the antimicrobial and proteolytic ability of NaOCl [14,22]. Another important issue is the strong demineralizing effect of EDTA. Prolonged exposure of root canal walls to this agent may lead to dentin alteration that negatively affects its mechanical properties [23,24], potentially influencing the resistance of the root to fatigue fractures [8,9].

The use of weaker chelating agents that do not decrease sodium hypochlorite activity and cause a milder erosive effect on dentin (compared with that of EDTA) has been previously studied [25,26,27]. Etidronic acid (1-Hydroxyethylidene-1,1-diphosphonic acid, HEDP; etidronate), a weak chelating agent, has shown promising results due to high smear layer removal ability, low erosive potential, and minor influence on the properties of NaOCl [14,22].

Although there are some systematic reviews on the antimicrobial and smear layer removal action of weak chelating agents including etidronate [28,29,30], very few studies have focused on the effect of these irrigants on the mechanical properties of root dentin [31,32].

A recent systematic review by Kumaresan et al. posed a research question regarding the effect of continuous chelation on the physicochemical properties of root canal dentin and concluded that continuous chelation had a minimal effect on dentin properties compared to sequential chelation. However, the authors did not provide a meta-analysis of the outcomes; moreover, some latest data on the topic were not included in their review.

The aim of this review was to qualitatively and quantitatively analyze the studies comparing the effect of continuous chelation (HEDP + NaOCl mixture) and conventional sequential protocol (EDTA and NaOCl) on the mechanical properties of dentin that influence root fracture resistance (namely, dentin erosion, microhardness, roughness, demineralization, and alteration in the Ca/P ratio).

## 2. Materials and Methods

This systematic review was registered on PROSPERO (CRD420250630663) and followed the PRISMA guidelines (PRISMA 2025 checklist can be found in Supplementary Materials).

### 2.1. Database Search

The PubMed and Scopus databases were searched for articles published between April 2009 and June 2026 (search date: 13 June 2026). Table 1 shows complete search queries in the aforementioned databases. No grey literature was included in this systematic review.

### 2.2. Eligibility Criteria

This systematic review was guided by the PICOS format as described below:

- Population (P)—root canal dentin of permanent human or animal teeth without previous history of endodontic treatment;
- Intervention (I)—continuous chelation (sodium hypochlorite and etidronate mixture);
- Comparison (C)—sequential irrigation protocol (sodium hypochlorite and EDTA);
- Outcome (O)—at least one of the properties that determine dentin mechanical properties including but not limited to root fracture resistance, dentin erosion, dentin demineralization, collagen damage, dentin microhardness, roughness, and chemical composition;
- Study design (S)—in vitro, ex vivo studies.

Only original articles written in English were included. We excluded studies that did not meet the PICOS criteria, along with other article types such as technical papers, clinical cases, literature reviews, guidelines, letters to the editor, and opinion articles.

### 2.3. Outcomes

The main outcome was root fracture resistance measured as a compressive load to fracture expressed in Newtons (N). Additional outcomes included other changes in the mechanical and structural properties of dentin such as erosion, chemical composition, surface roughness, and microhardness.

### 2.4. Data Screening and Extraction

Independent abstract screening was performed by 3 reviewers (MS, DS, and MP) at the first stage. The results of this screening were recorded using a table where reviewers described an article based on its eligibility (“relevant”/”irrelevant”), and divergent results were discussed until reaching a consensus. Subsequently, full texts of the articles were assessed and the data were extracted from eligible articles. The extracted data included authors, publication year, country, study design, sample characteristics (the type and number of teeth used, specimen preparation technique), intervention and control (types and concentrations of the irrigants, mode of their use), the outcomes and methods used to assess dentin properties, and the results. The data were independently extracted by 2 reviewers (KB and AM); the disagreements were resolved by a third researcher (NN).

### 2.5. Risk-of-Bias Assessment

Risk-of-bias assessment was performed independently by two reviewers (NN and AM) using the QUIN Tool [33]. A third reviewer (KB) was then consulted in cases of discrepancies.

Risk of bias was evaluated in the following domains: C1: Clearly stated aims/objectives; C2: Detailed explanation of sample size calculation; C3: Detailed explanation of sampling technique; C4: Details of the comparison group; C5: Detailed explanation of methodology; C6: Operator details; C7: Randomization; C8: Method of outcome measurement; C9: Outcome assessor details; C10: Blinding; C11: Statistical analysis; C12: Presentation of results.

The criteria for domain assessment were as follows:

2 points when adequately specified;

1 point when inadequately specified;

0 points when not specified.

The total score was calculated as the sum of the scores across all domains. Non-applicable domains were excluded from the calculation. The percentage risk of bias score (%RoB) was determined using the following formula:

Final Score = (Total score × 100)/(2 × number of criteria applicable).

The criteria for %RoB were as follows: “high risk” if %RoB is <50%; “medium risk” if %RoB is between 50 and 70%, and “low risk” if %RoB is >70%.

### 2.6. Strategy for Data Synthesis

A narrative and qualitative synthesis of the extracted data was performed. The data were entered into Microsoft Excel and synthesized separately for each property of root canal dentin including erosion, compressive fracture load, microhardness, chemical composition, roughness, collagen degradation, and flexural strength.

A quantitative synthesis for the extracted variables was performed if the included studies were sufficiently homogenous and provided sufficient data.

### 2.7. Statistical Analyses

Statistical analysis was conducted in R (v. 4.2.3) within the RStudio environment (v. 2023.03.0 + 386) using the “meta” package (version 2023.03.0 + 386). The meta-analysis was performed using a random-effects model. The standardized mean difference (SMD) was calculated as the primary effect size measure to compare continuous outcomes across studies using bias-corrected Hedges’ g statistic. Pooling of studies was conducted using the inverse variance method; the restricted maximum-likelihood estimator was used to calculate the between-study variance (τ^2^). Statistical heterogeneity was assessed using Cochran’s Q test and the I^2^ statistic.

For studies reporting continuous outcomes as pre-test and post-test values (namely, dentin microhardness studies), the mean change and the standard deviation of the change (SDchange) were calculated for each group. The mean change was defined as the difference between baseline and final measurements. Since the included studies did not report the correlation among measurements, a correlation coefficient (r) of 0.5 was assumed, following the recommendations of the Cochrane Handbook for Systematic Reviews of Interventions.

The SDchange was estimated using the following formula [34]:

$$SDchange=\;\sqrt[{2}]{\left({SDbaseline}^{2}+{SDfinal}^{2}-(2\times r\times SDbaseline\times SDfinal)\right)}$$

To assess potential publication bias, we used visual analysis of the contour-enhanced funnel plots followed by Egger’s linear regression test for funnel plot asymmetry [35].

The interrater reliability for risk of bias assessment was evaluated using the “irr” package. A weighted Cohen’s kappa was calculated to measure agreement between the two reviewers.

## 3. Results

### 3.1. Database Search and Selection Process

**Figure 1:**
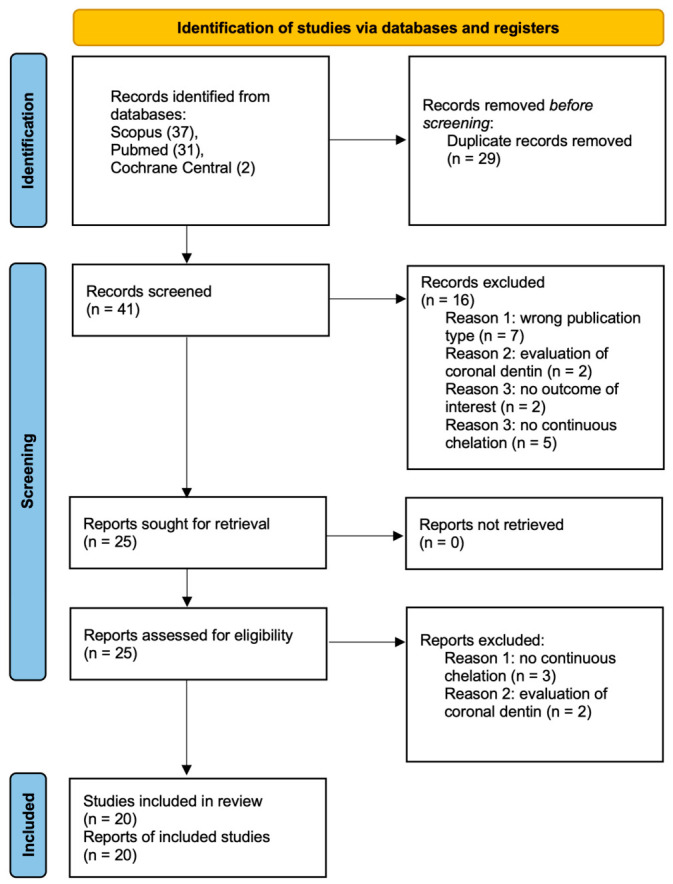
PRISMA flowchart diagram of the study selection procedure.

The PRISMA flow chart summarizes the study selection process (Figure 1). A total of 70 articles were identified from the initial search, 31 in PubMed, 37 in Scopus, and 2 in Cochrane central. After excluding 29 duplicates, 41 articles remained for the initial screening. After reading the titles and abstracts, we excluded 16 irrelevant studies. Twenty-five studies were included for full-text assessment, and of those, 5 were excluded after full-text analysis. One of the reasons for study exclusion at this step was the use of coronal dentin in the experiments [36,37,38,39], as mechanical properties of coronal and radicular dentin may vary due to structural differences [40]. The results of one study [41] were included partially because its authors used samples of coronal dentin for some investigations. Manual search in the reference lists of the included articles did not yield any additional studies. Hence, eighteen studies fulfilled the eligibility criteria for this review.

### 3.2. General Characteristics of the Included Studies

The general characteristics of the included articles were tabulated (Table 2). All of the studies included in this systematic review were ex vivo. The sample size varied between 1 [42] and 20 [43,44] teeth per group.

Most of the studies used human teeth including premolars [26,41,44,45,46,47,48,49,50,51,52], “single-rooted” teeth [25,27,43,53], “anterior” teeth [54,55], first incisors [54], and wisdom teeth [14]. Tartari and Wright used bovine incisors and mandibular canines, respectively [42,56]. Root canal instrumentation was performed with the following rotary systems: ProTaper (Dentsply Sirona, Tulsa, OK, USA) [25,26,42,44,46,47,49,51,56], Mtwo^®^ (VDW GmbH, Munich, Germany) [14,48], Reciproc (VDW GmbH, Munich, Germany) [45], AF Blue (Fanta Dental Materials Co., Shanghai, China) [41], ProFile (Dentsply Maillefer) [50], and WaveOne Gold (Dentsply Sirona, Tulsa, OK, USA) [52]. Continuous chelation was carried out with etidronic acid at final concentrations of 9% [14,25,26,27,44,45,46,47,48,49,50,51,52,53,54,55], 15% [14], 18% [14,41,42,53,55], or 7.7% [56] and NaOCl at final concentrations of 2.5% [27,44,45,46,47,48,49,51,53,54], 5% [42,43,52,53,54,56], 3% [14,25,26,41], or 1–2% [50]. The majority of the studies used EDTA at a concentration of 17% [14,25,26,27,41,42,43,44,45,46,47,48,49,50,51,52,53,54,55,56], while one study used it at a concentration of 12.5% [41]. The total irrigation time ranged from 5 to 62.5 min, with irrigant volumes ranging from 5 mL [42] to 18.5 mL [56]. Final irrigation took from 1 to 5 min with total irrigating solutions volumes ranging from 1 mL [42] to 10 mL [25]. Some of the protocols implied ultrasonic or sonic activation of the irrigants [26,46,48].

**Table 2:** Characteristics and main findings of the included studies.

Author, Reference	Outcome	Assessment Method	Irrigation Protocols		Specimens’ Characteristics	Findings (HEDP vs. EDTA)
			Intervention	Control		
Ballal et al. 2024 [44]	Fracture resistance	Static load in a universal testing machine after thermo-mechanical aging	2.5% NaOCl + 9% HEDP 15 mL 6 min	2.5% NaOCl + 17% (10 mL, 5 min), EDTA (5 mL, 1 min)	Roots of human single-rooted mandibular first premolars (*n* = 15), canal size–ProTaper F3	The HEDP group had higher fracture resistance
Yeter et al. 2024 [45]	Fracture resistance	Static load in a universal testing machine	2.5% NaOCl + 9% HEDP 6 mL ? min	2.5% NaOCl (3 mL) + 17% EDTA (3 mL, 1 min)	Roots of human mandibular premolars (*n* = 11) Canal size Reciproc 50	No differences
Wright et al. 2021 [56]	Fracture resistance	Static load in a universal testing machine	2.5% NaOCl + 7.7% HEDP 18.5 mL 62.5 min	5% NaOCl (16.5 mL, 60.5 min) + 17% EDTA (2 mL 2 min)	Roots of bovine teeth (*n* = 15) Canal size ProTaper Next X5	No differences
Ulusoy et al. 2021 [46]	Fracture resistance	Static load in a universal testing machine	NaOCl + 9% HEDP 6 mL 2 min With and without PUI	2.5% NaOCl (3 mL 1 min) + 17% EDTA or 2.5% (3 mL 1 min) With and without PUI	Roots of human single-rooted mandibular premolars (*n* = 10); 3 different wall thicknesses (0.75, 1.5, and 2.25 mm) Canal size ProTaper Universal F3	The HEDP group had higher resistance to fracture
Gonzalez et al. 2020 [48]	Fracture resistance	Static load in a universal testing machine	2.5% NaOCl + 9% HEDP 3 mL 1 min	2.5% NaOCl (4.5 mL 2 min) + 17% EDTA (3 mL 3.5 min)	Roots of human premolars (*n* = 16) Canal size Mtwo 40.04	No differences
Lantigua Domínguez et al. 2018 [49]	Fracture resistance	Static load in a universal testing machine	2.5% NaOCl + 9% HEDP 12 mL 25 min	2.5% NaOCl (? mL 25 min) + 17% EDTA (3 min)	Roots of human premolars with a single root canal (*n* = 10) Canal size ProTaper Next X3	The HEDP group had lower fracture resistance
Ballal et al. 2024 [44]	Microhardness	Knoop hardness tester	2.5% NaOCl + 9% HEDP 15 mL 6 min	2.5% NaOCl + 17% (10 mL, 5 min), EDTA (5 mL, 1 min)	Root segments (*n* = 10) of human single-rooted mandibular first premolars	The HEDP group had higher microhardness values
Hazar et al. 2025 [54]	Microhardness	Automatic turret microhardness tester	2.5% NaOCl + 9% HEDP 32 mL 20 min (immersion)	2.5% NaOCl (32 mL 20 min) + 17% EDTA (4 mL 2 min)	Human first maxillary incisors (*n* = 10)	The HEDP group had smaller changes in microhardness values
Tartari et al. 2013 [53]	Microhardness	Microhardness tester	2.5% NaOCl + 9% HEDP 40 mL 30 min (immersion)	2.5% NaOCl (33 min) + 17% EDTA (3 min)	Human single-rooted teeth (*n* = 9), three root thirds	No significant differences
Elika et al. 2021 [43]	Microhardness	Vickers microhardness teste	5% NaOCl + 18% HEDP (? mL 15 min) immersion	5% NaOCl + 17% (? mL, 7.5 min) EDTA (? mL, 7.5 min) immersion	Human single-rooted teeth (*n* = 20)	The HEDP group had smaller changes in microhardness values
Ulusoy et al. 2020 [47]	Nanohardness	Nanoindenter with a Berkovich tip	2.5% NaOCl + 9% HEDP 6 mL 2 min	2.5% NaOCl + 17% (3 mL, 1 min) EDTA 3 mL, 1 min)	Dentin blocks from human single-rooted mandibular premolars (*n* = 10)	The HEDP group had greater changes in microhardness values
Mikheikina et al. 2024 [14]	Dentin erosion	Scanning electron microscopy	or 3% NaOCl + 9, 15, or 18% HEDP 15 mL ? min	3% NaOCl + 17% (15 mL ? min) EDTA (2 mL ? min)	Human wisdom teeth (*n* = 10) three root thirds	HEDP had similar or smaller erosive effect
Zarean et al. 2023 [26]	Dentin erosion	Micro-computed tomography	3% NaOCl + 9% HEDP (8 mL 2 min), PUI	3% NaOCl + 17% (13 mL ? min) EDTA (8 mL 2 min), PUI	Human upper premolars (*n* = 10) three root thirds	HEDP had smaller erosive effect
Kfir et al. 2020 [25]	Dentin erosion	Scanning electron microscopy	or 3% NaOCl + 9% HEDP 10 mL 5 min	3% NaOCl + 17% (8 mL, 4.5 min) EDTA (2 mL, 0.5 min)	Human single-rooted teeth (*n* = 20) three root thirds	No differences
Lima Nogueira et al. 2018 [27]	Dentin erosion	Scanning electron microscopy	2.5% NaOCl +9% HEDP ? mL 30 min	2.5% NaOCl (? mL, 30 min), 17% EDTA (? mL, 30 min)	Human single-rooted teeth (*n* = 5)	HEDP had greater erosive effect
Tartari et al. 2023 [42]	Dentin erosion	Scanning electron microscopy	2.5% NaOCl + 9% HEDP ? mL 5 min	2.5% NaOCl (? mL 5 min) + 17% EDTA (? mL, 1 min)	Bovine incisors with completely formed roots (*n* = 1)	HEDP had smaller erosive effect
Rath et al. 2020 [41]	Dentin erosion	Scanning electron microscopy	or 5% NaOCl + 18% HEDP ? mL ? min	5% NaOCl + 17% EDTA ? mL ? min	Single-rooted human mandibular premolars (*n* = 8)	No differences
Ulusoy et al. 2020 [47]	Dentin erosion	Scanning electron microscopy	2.5% NaOCl + 9% HEDP 6 mL 2 min	2.5% NaOCl + 17% (3 mL, 1 min) EDTA 3 mL, 1 min)	Human single-rooted mandibular premolars (*n* = 10)	HEDP had greater erosive effect
Khairy et al. 2026 [51]	Dentin erosion	Scanning electron microscopy	2.5% NaOCl + 9% HEDP 7.5 mL 5 min	2.5% NaOCl + 17% (? mL, 4 min) EDTA (? mL, 1 min)	Human mandibular premolar (*n* = 12)	HEDP had smaller erosive effect only in the apical third
Bhandari et al. 2026 [52]	Dentin erosion	Scanning electron microscopy	5.25% NaOCl + 9% HEDP ? mL 10 min	5.25% NaOCl +17% (? mL, 5 min) EDTA (? mL, 5 min)	Human premolars (*n* = 3)	HEDP had smaller erosive effect
Lottanti et al. 2009 [50]	Chemical composition	Atomic absorption spectroscopy and scanning electron microscopy in backscatter mode	or 1% NaOCl + 9% HEDP ? mL 18 min	1% NaOCl + 17% (10 mL, 15 min) EDTA (5 mL, 3 min)	Human single-rooted premolars (*n* = 12)	HEDP removed less calcium from the canal system; less pronounced decalcifications in the specimens irrigated with HEDP
Lima Nogueira et al. 2018 [27]	Chemical composition	Energy dispersive X-ray spectrometry and X-ray diffraction	2.5% NaOCl + 9% HEDP ? mL 30 min	2.5% NaOCl (? mL, 31 min), 17% EDTA (? mL, 30 min)	Single-rooted human teeth (*n* = 5)	No differences in the content of K and Ca/P ratio; the content of Mg, P, and Ca was higher in the HEDP group
Rath et al. 2020 [41]	Chemical composition	Transmission electron microscopy	or 5% NaOCl + 18% HEDP ? mL ? min	5% NaOCl + 17% EDTA ? mL ? min	Dentin blocks from single-rooted human mandibular premolars (*n* = 12)	The HEDP group had less exposed and demineralized collagen fibers
Bhandari et al. 2026 [52]	Chemical composition	Energy dispersive X-ray spectrometry and Fourier infrared spectroscopy	5.25% NaOCl + 9% HEDP ? mL 10 min	5.25% NaOCl + 17% (? mL, 5 min) EDTA (? mL, 5 min)	Human premolars (*n* = 3)	HEDP mainly caused collagen denaturation; EDTA–both demineralization and denaturation of collagen
Tartari et al. 2013 [55]	Dentin roughness	Portable digital roughness tester	2.5% NaOCl + 9% HEDP ? mL 30 min	2.5% NaOCl + 17% (? mL, 30 min) EDTA (? mL, 3 min)	Human anterior teeth (halves) (*n* = 10)	The HEDP group had greater increase in surface roughness
Ballal et al. 2024 [44]	Flexural strength	Three-point flexure test using the Instron universal testing machine	2.5% NaOCl + 9% HEDP 15 mL 6 min	2.5% NaOCl + 17% (10 mL, 5 min), EDTA (5 mL, 1 min)	Dentin bars (1 × 1 × 10 mm) made of human single-rooted mandibular first premolars (*n* = 10)	The HEDP group had higher flexural strength values

NaOCl—sodium hypoclorite, EDTA—ethylenediaminetetraacetic acid, HEDP—1-Hydroxyethylidene-1,1-diphosphonic acid, PUI—passive ultrasonic irrigation, ?—information is not specified in the article.

Among the included studies, nine evaluated dentin erosion [14,25,26,27,41,42,47,51,52], six tested fracture resistance [44,45,46,48,49,56], five studies focused on microhardness [43,44,47,53,54], and four conducted chemical composition analysis [27,41,50,52]. Dentin roughness [55] and flexural strength [44] were studied in one study each. All-in-all, 29 sets of findings from 20 studies were analyzed, encompassing comparisons between sequential chelation using EDTA and continuous chelation with etidronate-NaOCl mixture.

### 3.3. Risk of Bias Assessment

**Figure 2:**
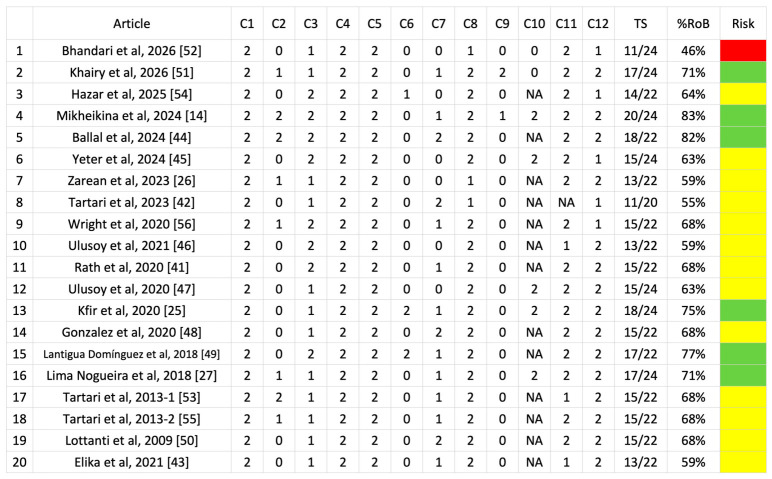
Quality assessment of the selected studies (the QUIN tool for assessing risk of bias). C1: Clearly stated aims/objectives; C2: Detailed explanation of sample size calculation; C3: Detailed explanation of sampling technique; C4: Details of the comparison group; C5: Detailed explanation of methodology; C6: Operator details; C7: Randomization; C8: Method of outcome measurement; C9: Outcome assessor details; C10: Blinding; C11: Statistical analysis; C12: Presentation of results. TS: Total score. A criterion was excluded from the TS calculation when it was not applicable (NA). Green, yellow, and red indicate low, medium, and high risk of bias, respectively.

Six of the included studies were considered to have a low risk of bias, 13 of them were classified as having a medium risk of bias, while one study had a high risk of bias (Figure 2). The inter-examiner reliability score for the risk of bias between the authors (N.N and K.B) was 0.993, showing that the agreement was almost perfect (*p* < 0.01).

### 3.4. Qualitative Synthesis

Eight outcomes from 20 studies were scrutinized for qualitative synthesis.

#### 3.4.1. Fracture Resistance

Six studies investigated the fracture resistance of teeth treated with NaOCl mixed with 7.7% [56] or 9% etidronate [44,45,46,48,49] or with 17% EDTA and NaOCl sequentially. These studies used 2.5% NaOCl and 17% EDTA. The study by Wright et al. was the only one to assess the fracture resistance of bovine teeth, while other studies assessed the properties of human teeth [56]. The total number of specimens was 154. ProTaper systems were the most commonly used for root canal instrumentation [44,45,46,49,56], with an ISO 30 apical diameter being the most frequent choice [44,45,46,49]. Gonzalez et al. instrumented root canals with the Mtwo system (40.04) [48]. Fracture resistance was tested either in teeth with non-obturated canals [48,49,56] or after root canal obturation [44,45,46]. Only one study used thermo-mechanical cycling before subjecting the specimen to static loading [44]. The volumes of the etidronate–NaOCl mixture ranged from 3 mL to 18.5 mL. Two studies activated irrigant solutions with ultrasonic [46] or sonic [48] devices.

The included studies followed different durations of treatment. Moreover, in some studies, the etidronate–NaOCl mixture was used during the final irrigation step rather than throughout the entire root canal preparation phase [45,46,48]. While the etidronate–NaOCl mixture is intended for continuous use throughout instrumentation, the contact time for EDTA (as a strong chelator) should be restricted to 1–2 min in accordance with existing evidence [57,58]. When the control protocols limited EDTA use to 1–2 min, continuous chelation demonstrated results similar to or better than the sequential protocol [45,46,48,56]. Three of the studies found no difference in fracture resistance between the continuous and sequential protocols [45,48,56], while Ulusoy et al. (employing a 1 min EDTA treatment) reported that etidronate had a smaller detrimental effect on dentin than EDTA [46]. Ballal et al. reported similar results; although the EDTA exposure time was not reported, the continuous chelation group showed significantly higher fracture resistance than the sequential group [44]. The study by Lantigua Domínguez et al. was the only one that favored the sequential protocol, reporting lower fracture resistance after continuous chelation; notably, this study utilized the longest EDTA exposure (3 min) [49]. In summary, under clinically realistic EDTA exposure time (1–2 min) continuous chelation had the same or greater fracture resistance as the sequential protocol, while the only unfavorable finding was registered after a prolonged exposure not typical for clinical practice.

#### 3.4.2. Erosion

Nine studies assessed dentin erosion after different irrigation protocols. Eight used human teeth and one used bovine teeth; importantly, the study by Tartari et al., which used bovine teeth, was based on a single specimen (*n* = 1) [42], so its result should be weighted accordingly. Five studies assessed erosion in different root thirds [14,25,26,27,47], one evaluated only the middle third, and Rath et al. did not specify the area analyzed [41]. Etidronate concentrations were 9% [14,25,26,27,47], 15% [14], or 18% [14,41,42].

The EDTA contact time differed widely: 0.5 min in the study by Kfir et al. [25], 1 min in the studies by Ulusoy et al. [47], Tartari et al. [42], Khairy et al. [51], 2 min in the study by Zarean et al. (with passive ultrasonic activation) [26], 5 min in the study by Bhandari et al. [52], and 30 min in the study by Lima Nogueira et al. [27]. Mikheikina et al. [14] and Rath et al. [41] did not report the irrigation time. Most studies assessed erosion using SEM [14,25,27,41,42,47], while Zarean et al. used micro-CT [26]. Erosion was scored either qualitatively [41,42] or quantitatively using the Torabinejad [14,27,47] or Kaya [25] scales.

Among the studies using short EDTA contact times, continuous chelation was generally better. Zarean et al. (2 min, micro-CT) reported less erosion with HEDP [26], and Kfir et al. (0.5 min, SEM) [25] found no difference between protocols. Tartari et al. observed lower erosion with HEDP in a single bovine specimen [42]. Similarly, Khairy et al. found a reduced erosive effect with HEDP, which was confined to the apical third [51]. The only short-protocol study reporting a greater detrimental effect of HEDP was that by Ulusoy et al. (2 min) [47]. Among the prolonged-exposure studies, Lima Nogueira et al. (30 min) reported greater erosion with HEDP [27]. Conversely, Bhandari et al. (5 min) appeared to favor HEDP [52]; however, this finding was mentioned indirectly and was based on three specimens. In the two studies that did not report the irrigation time, HEDP produced similar or less erosion (Mikheikina et al. [14]) or no difference (Rath et al. [41]). Overall, most studies reported similar or lower dentin erosion with continuous chelation, and the two findings unfavorable to HEDP came from a single research group [47] and from the longest exposure [27].

#### 3.4.3. Microhardness and Nanohardness

A total of five studies were included in the qualitative synthesis; of these, four evaluated microhardness and one evaluated nanohardness after sequential and continuous irrigating protocols. The studies utilized human teeth, with a total number of specimens amounting to 118. Most of the studies used 9% etidronate and 2.5% NaOCl [44,47,53,54], while one study [43] employed 18% and 5% solutions, respectively. The concentration of EDTA was 17% in all studies.

Two studies assessed hardness after conventional in-canal irrigation with comparatively short, clinically relevant EDTA contact times: Ballal et al. applied 5 mL over 1 min [44], and Ulusoy et al. applied 3 mL over 1 min [47]. In the remaining three studies the specimens were immersed in the solutions for prolonged periods, which differed from clinical irrigation protocols: 7.5 min in Elika et al. [43], 2 min in Hazar et al. [54], and 3 min in Tartari et al. [53]. The measurement approaches also differed: Ballal et al. assessed Knoop hardness at 50 μm from the canal wall rather than at the surface [44], and Ulusoy et al. measured nanohardness by nanoindentation on dentin blocks rather than microhardness [47].

Across the studies with prolonged immersion (3–7.5 min), continuous chelation was equally or less detrimental than the sequential protocol. Specifically, HEDP produced a smaller reduction in microhardness than EDTA in the study by Elika et al. (7.5 min) [43]. In the study by Tartari et al. (3 min), there were no substantial differences between the groups, although the authors did not provide the statistical analysis of intergroup differences between irrigation protocols [53]. Among the studies using shorter EDTA treatment times, Ballal et al. (1 min) and Hazar et al. (2 min) also reported higher microhardness values in the HEDP group [44,54]. The only finding unfavorable to continuous chelation was reported by Ulusoy et al., who found a greater reduction in hardness with NaOCl + HEDP [47]; however, this was a nanohardness measurement performed on dentin blocks rather than a microhardness measurement. Overall, the reduction in dentin microhardness with continuous chelation was equal to or smaller than that with the sequential EDTA protocol, regardless of the irrigation duration.

#### 3.4.4. Influence on Root Canal Dentin Composition

Three studies analyzed the effect of the irrigation protocols on the chemical composition of root canal dentin (*n* = 37). The methods used were energy-dispersive X-ray spectrometry and X-ray diffraction [27], atomic absorption spectroscopy with scanning electron microscopy in backscatter mode [50], and energy-dispersive X-ray spectrometry combined with Fourier-transform infrared spectroscopy [52]. All studies used 17% EDTA and 9% etidronate, while the NaOCl concentration was 1% [50], 2.5% [27], or 5.25% [52]. All three studies applied the solutions for prolonged periods—30 min [27], 5 min [52], and 3 min [50]—exceeding the recommended contact times for clinical irrigation. According to Lima Nogueira et al., there were no significant differences between the EDTA and HEDP groups in the diffractograms, potassium content, or the Ca/P ratio; however, the levels of magnesium, phosphorus, and calcium were significantly lower in the EDTA group [27]. Lottanti et al. found that the protocols using EDTA eluted almost twice as much calcium from the canal system compared to etidronic acid, and SEM in backscatter mode demonstrated more pronounced decalcification in specimens irrigated with EDTA [50]. In line with this, Bhandari et al. reported that EDTA caused both demineralization and collagen denaturation, whereas the HEDP mixture produced mainly collagen denaturation with little demineralization [52]. Overall, these results suggest that the sequential protocol exhibits a stronger decalcifying potential than continuous chelation; however, the findings by Bhandari et al. indicate that etidronate may still affect the organic (collagen) component of dentin [52].

#### 3.4.5. Roughness

Only one study reported on human radicular dentin roughness (*n* = 18) [55]. The authors treated the surface of the root canals with either 9% etidronate + 2.5% NaOCl or 2.5% NaOCl and 17% EDTA for 30 min. The arithmetic roughness average (Ra) was measured with a portable digital roughness tester [55]. The researchers [55] concluded that continuous chelation with HEDP produced a greater increase in surface roughness compared to sequential chelation with EDTA.

#### 3.4.6. Flexural Strength

Ballal et al. reported on the flexural strength of dentin treated with the studied protocols [44]. The authors compared the effect of sequential irrigation with 2.5% NaOCl and continuous irrigation with 17% EDTA and 9% etidronate /2.5% NaOCl mixture. They found that flexural strength in the etidronate group was significantly higher than that observed in the EDTA group.

### 3.5. Quantitative Synthesis (Meta-Analysis)

Eight of the included studies were suitable for meta-analysis. It was performed separately for the fracture resistance outcome. The rationale for including studies in the meta-analysis was to maintain methodological consistency and warrant data compatibility for quantitative synthesis.

The studies assessing dentin microhardness and erosion implemented heterogeneous approaches to evaluate the effects of the chelating protocols and did not provide sufficient quantitative data to be analyzed. Furthermore, there were not enough studies on dentin roughness, chemical composition, collagen degradation, and flexural strength to perform a meta-analysis. Therefore, only a qualitative synthesis was possible for the aforementioned outcomes.

#### Fracture Resistance

The data from five studies on fracture resistance were pooled for meta-analysis [44,45,46,48,49]. The study by Wright et al. was excluded as the authors used bovine teeth for the experiments. Two studies were classified as having a low risk of bias [44,49], while three studies were classified as having a medium risk [45,46,48].

**Figure 3:**
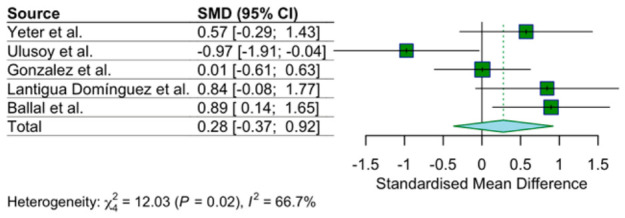
Forest plot of the meta-analysis evaluating fracture load [44,45,46,48,49].

A meta-analysis of the included studies (*n* = 94) yielded the pooled standardized mean difference SMD of 0.28 (−0.37; 0.92) (Figure 3). Although a small positive trend was observed, the result did not reach the level of statistical significance (*z* = 0.84, *p* = 0.3984). We found substantial heterogeneity (I^2^ = 66.7%, τ^2^ = 0.037), suggesting that the findings were substantially inconsistent among the included studies.

On the contour-enhanced funnel plot, the studies are located in the white and grey (non-significant) regions (*p* > 0.1), which is consistent with the individual study results. The studies are positioned in the lower half of the plot (a higher standard error), indicating relatively small sample sizes and lower precision. Nevertheless, the publication bias analysis should be interpreted with caution, as it included fewer than 10 studies.

**Figure 4:**
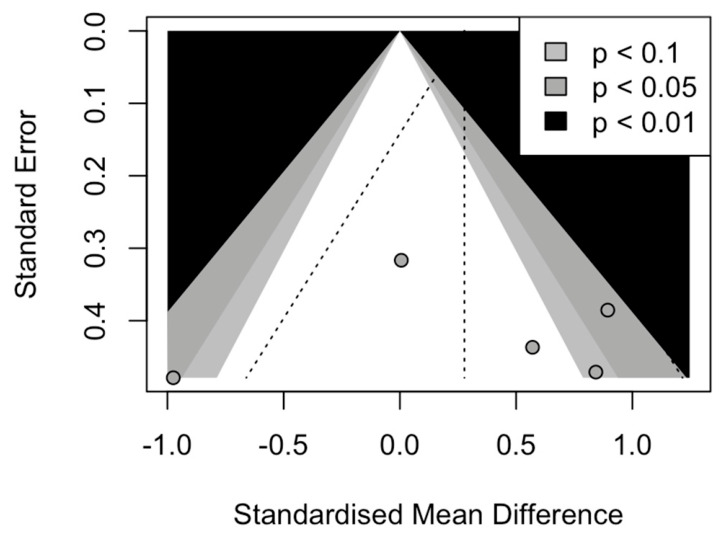
Contour-enhanced funnel plot for the assessment of publication bias. The standardized mean difference (SMD) is plotted on the *x*-axis, while the *y*-axis represents the standard error. The vertical dashed line represents the pooled effect size (SMD = 0.26).

The linear regression test for funnel plot asymmetry yielded a *t*-value of −0.04 with a *p*-value of 0.9717 (Figure 4). This indicates a highly symmetrical distribution of studies around the pooled effect size. Since the *p*-value is well above the 0.05 threshold, we found no evidence of significant publication bias in this meta-analysis.

## 4. Discussion

This systematic review and meta-analysis compared the effects of continuous chelation using etidronic acid against the conventional sequential application of NaOCl and EDTA on the mechanical properties of root dentin. The main finding from the analyzed in vitro studies was that while there was no statistically significant difference in fracture resistance between the two irrigation protocols, the qualitative synthesis revealed that HEDP seems to have an equal or less detrimental effect on dentin in terms of erosion, chemical composition, and dentin microhardness compared to EDTA. To the best of our knowledge, this is the first systematic review to include a meta-analysis specifically focused on mechanical outcomes, such as fracture resistance, when comparing continuous chelation and sequential irrigation protocols.

When comparing mechanical properties of dentin after treatment with chelating agents, it is important to account for the exposure time. While the etidronate–NaOCl mixture is meant to be used continuously throughout the instrumentation procedure, the exposure time to EDTA (as a strong chelator) must be restricted to 1–2 min, in line with existing recommendations [57,58]. Evidence suggests that a 1–2 min EDTA application is effective enough in removing the smear layer from all root canal thirds [57,58], while a 10 min EDTA treatment has been associated with excessive damage to peritubular and intertubular dentin [58]. Therefore, the results of the included studies must be interpreted in light of these exposure variations. Most of the analyzed studies used clinically appropriate EDTA exposure times (1–3 min); however, some studies used an unnecessarily prolonged exposure: 5 min [52], 7.5 min [43], 30 min [27].

The primary outcome, root fracture resistance, may be regarded as an important measure of a tooth’s capacity to withstand occlusal function over time. This meta-analysis, which pooled data from five studies, did not find a statistically significant difference between the HEDP and EDTA groups (*p* = 0.3984). Although a small positive trend favored the etidronate protocol, the result suggests that, based on current in vitro evidence, the use of continuous chelation does not compromise the resistance of the root to vertical fracture irrespective of the exposure time. This finding is consistent with the qualitative results from most of the included studies, where four out of six experiments demonstrated no differences between the protocols. The conflicting results from the remaining two studies, one favoring HEDP [46] and the other favoring EDTA [49], highlight the methodological variability that may have influenced these outcomes. The substantial heterogeneity and low risk of publication bias in the meta-analysis, as well as mainly medium bias according to the QUIN tool, support the insignificance of differences between the protocols.

The qualitative synthesis of other mechanical properties confirmed that HEDP is a less aggressive chelating agent than EDTA. The studies assessing dentin microhardness favored continuous chelation with HEDP. Microhardness can be considered an important surrogate outcome reflecting the degree of dentin demineralization [59]. The less pronounced reduction in microhardness observed with HEDP is likely due to its weaker chelating nature compared to the more aggressive 17% EDTA. This finding is in line with several other systematic reviews which concluded that etidronate has a milder demineralizing effect [30,60].

These findings should be interpreted with caution, as the included studies exhibited substantial methodological heterogeneity, which precluded a meta-analysis. In contrast, Ulusoy et al. found a greater reduction in hardness with NaOCl + HEDP [47]; however, it is important to note that they assessed nanohardness rather than microhardness, meaning these results cannot be directly compared. Further research is required to confirm whether continuous chelation differs from the sequential protocol in its effect on this property of dentin.

In the studies assessing dentin erosion, the EDTA exposure duration differed widely, including unnecessarily prolonged contact times exceeding 5 min [52] and 30 min [27]. Also, the treatment time was not reported in two studies [14,41]. The majority of the studies on dentin erosion found that HEDP caused similar or lower levels of surface damage. This is an important advantage, as excessive erosion can weaken the dentin structure and create surface irregularities that can reduce its resistance to crack initiation and propagation [8,9]. The two findings unfavorable to HEDP came from a single research group [47] and from the study with the longest exposure time [27].

Chemical analysis showed that EDTA eluted significantly more calcium from the dentin, confirming its stronger demineralizing potential [27,50]. Furthermore, studies on collagen degradation [41,52] and flexural strength [44] favored the HEDP protocol, showing less damage to the organic matrix and higher flexural strength values, respectively. The only contradictory finding came from a single study on surface roughness, which reported a greater increase with HEDP [53].

Our findings align with the broader literature. A recent scoping review by La Rosa et al. concluded that continuous chelation was comparable or superior to sequential irrigation for smear layer removal and antimicrobial activity, while causing less dentin erosion [60]. Another systematic review by Vidal-Montolío et al. focused on smear layer removal and also found HEDP to be a viable alternative to EDTA [29]. The present review builds upon this evidence and provides a quantitative synthesis of the findings on mechanical properties, supporting the use of HEDP as a dentin-preserving chelator.

Our findings are also consistent with the conclusions of a recent systematic review by Elfarraj et al., who analyzed the effects of endodontic irrigants on dentin composition using Fourier-transform infrared spectroscopy [32]. Their review of 30 studies showed that NaOCl mainly affects the organic component of dentin, while EDTA primarily targets the inorganic component, with stronger effects at longer exposure times and higher concentrations. These observations support our qualitative findings that EDTA has a stronger demineralizing potential than HEDP as demonstrated by the greater calcium loss reported by Lottanti et al. and the more pronounced erosion seen in the majority of included studies. Furthermore, Elfarraj et al. highlighted that the protocol of application, rather than the irrigant type alone, plays a significant role in determining the extent of the chemical damage to dentin. This is particularly relevant to our review, as the continuous chelation protocol with HEDP differs from sequential EDTA use not only regarding the chelating agent itself but also in the duration of dentin treatment. Taken together, the spectroscopic evidence from Elfarraj et al. and the mechanical data from our review suggest that the choice of a chelating agent and irrigation protocol impact dentin integrity, underlining the importance of less damaging alternatives, such as etidronic acid, in clinical practice.

A focused review by Rath et al. studied the effects of different irrigants on dentin composition and mechanical properties and concluded that all the assessed irrigation sequences decreased dentin flexural strength and microhardness [41]. At the same time, the authors identified only one study [61] that investigated the effect of continuous chelation protocol on the mechanical properties of dentin; moreover, in that study NaOCl and HEDP were applied sequentially rather than as a mixture. The present systematic review includes newer articles and a greater number of outcomes related to the mechanical properties of dentin. Despite the possible adverse effects of any irrigation protocol on dentin properties, our review supports the use of etidronic acid as a less aggressive alternative to EDTA.

A recently published systematic review by Kumaresan et al. addressed a similar research question by evaluating the effect of continuous chelation on the physicochemical properties of root canal dentin [31]. Their review included eight in vitro studies and concluded that continuous chelation had a minimal effect on dentin properties compared to sequential chelation. While our findings are generally consistent with this conclusion, the present review differs from that of Kumaresan et al. by including a substantially larger number of studies (20 vs. 8) and performing a meta-analysis specifically for the fracture resistance outcome. This approach provided a more precise estimate of the effect: specifically, we demonstrated that the difference in fracture resistance was not statistically significant. Overall, the present review builds upon and extends the work of Kumaresan et al. by providing a broader evidence base, a quantitative synthesis, and a more detailed analysis of the individual mechanical outcomes.

Several limitations of this review must be acknowledged. First, all included studies were conducted ex vivo. These laboratory models cannot fully replicate the complex biological and anatomic environment of the oral cavity, thereby impeding the extrapolation of results to clinical situations. Second, substantial methodological diversity and heterogeneity were observed across the studies regarding irrigant concentrations, application times, and activation methods. This dissimilarity compromises direct comparisons of the studies and reduces the strength of the quantitative synthesis. Third, the overall quality of the included studies was moderate, with most having a medium risk of bias according to the QUIN tool [33]. Common shortcomings included a failure to report sample size calculations and a lack of sample randomization. These factors may increase the risk of bias and reduce the overall strength of the evidence.

Despite the aforementioned limitations, the existing in vitro evidence suggests that the continuous chelation protocol with HEDP is comparable to the sequential use of EDTA in terms of root fracture resistance, microhardness, and other mechanical properties of root dentin. This is particularly important in the context of minimally invasive endodontics, where preserving the integrity of the tooth structure is a primary goal. However, further research is essential. Well-designed randomized controlled trials are required to determine if the benefits in mechanical properties translate into improved clinical outcomes, such as increased tooth survival and reduced incidence of vertical root fractures. Future laboratory studies should also use standardized protocols to reduce heterogeneity and improve the quality of results.

## 5. Conclusions

Based on the analyzed in vitro studies, continuous chelation using a mixture of NaOCl and etidronic acid appears to be a less aggressive and potentially safer irrigation protocol compared to the conventional sequential use of NaOCl and EDTA, although clinical validation is still required.

## Figures and Tables

**Table 1:** **Complete search queries in databases**.

Pubmed	(“etidronic acid”[MeSH Terms] OR “etidronic acid”[tw] OR “HEBP”[tw] OR “HEDP”[tw] OR “etidronate”[tw] OR “1-hydroxyethylidene-1,1-diphosphonic acid”[tw] OR “continuous chelation”[tw]) AND (“sodium hypochlorite”[MeSH Terms] OR “sodium hypochlorite”[tw] OR “NaOCl”[tw]) AND (“edetic acid”[MeSH Terms] OR “EDTA”[tw] OR “ethylenediaminetetraacetic acid”[tw] OR “sequential chelation”[tw]) AND (“Tooth fractures”[MeSH Terms] OR “root fracture*”[tw] OR “dentin microhardness”[tw] OR “fracture resistance”[tw] OR “dentin roughness”[tw] OR “erosive effect*”[tw] OR “deminerali*”[tw] OR “Ca/P ratio”[tw] OR “decalc*”[tw] OR “erosion”[tw] OR “collagen degradation”[tw])
Scopus	(TITLE-ABS-KEY (“etidronic acid” OR “HEBP” OR “HEDP” OR “etidronate” OR “continuous chelation”) AND TITLE-ABS-KEY (“sodium hypochlorite” OR “NaOCl”) AND TITLE-ABS-KEY (“edetic acid” OR “EDTA” OR “ethylenediaminetetraacetic acid” OR “sequential chelation”) AND TITLE-ABS-KEY (“Tooth fracture*” OR “root fracture*” OR “dentin microhardness” OR “fracture resistance” OR “dentin roughness” OR “erosive effect*” OR “demineralisation” OR “Ca/P ratio” OR “decalc*” OR “erosion” OR “collagen degradation”))
Cochrane central	([mh “Etidronic Acid”] OR “etidronic acid” OR “HEBP” OR “HEDP” OR “etidronate” OR “1-hydroxyethylidene-1,1-diphosphonic acid” OR “continuous chelation”):ti,ab,kw AND ([mh “Sodium Hypochlorite”] OR “sodium hypochlorite” OR “NaOCl”):ti,ab,kw AND ([mh “Edetic Acid”] OR “EDTA” OR “ethylenediaminetetraacetic acid” OR “sequential chelation”):ti,ab,kw AND ([mh “Tooth Fractures”] OR (root NEXT fracture*) OR (tooth NEXT fracture*) OR “dentin microhardness” OR “fracture resistance” OR “dentin roughness” OR (erosive NEXT effect*) OR deminerali* OR “Ca/P ratio” OR decalc* OR “erosion” OR “collagen degradation”):ti,ab,kw

## Data Availability

No new data were created or analyzed in this study. Data sharing is not applicable to this article.

## Supplementary Materials

The following supporting information can be downloaded at: https://www.mdpi.com/article/10.3390/dj14070450/s1, Table S1: PRISMA 2020 checklist. Ref. [62] is cited in Supplementary Materials.
